# Fibrous contextual embodiments formed by task-invariant continuous blow spinning in dynamic environments

**DOI:** 10.3389/frobt.2026.1736915

**Published:** 2026-03-25

**Authors:** Marie Vihmar, Roman Boiko, Kadri-Ann Pankratov, Friedrich Kaasik, Kärt Ojavee, Indrek Must

**Affiliations:** 1 IMS Lab, Institute of Technology, University of Tartu, Tartu, Estonia; 2 Department of Design, Estonian Academy of Arts, Tallinn, Estonia

**Keywords:** bio-inspired robotics, contextual embodiments, dynamic environments, embodied AI, embodied intelligence, growing robots, melt blow spinning, soft robotics

## Abstract

Navigating dynamic environments is challenging for robots. While data-intensive control can guide high-level decisions, the complexity of typical natural settings, dynamic in particular, motivates offloading part of intelligent behavior to the robot’s morphology. Here, we introduce an *in situ*, model-free melt-blown process that creates unsupported thermoplastic webs via direct environmental interaction, enabling *ad hoc* robotic embodiments. When spun on moving supports, fiber elasticity causes support motion to align fibers and homogenize the web rather than breaking it, yielding ∼60% reversible strain and stretch to ∼1,000% at ultimate rupture. We illustrate task-invariant fiber spinning in several task contexts relevant to complex environments: creating mobility pathways in moving environments, immobilizing moving objects, sampling discrete materials, and capturing resources (oil-soaked sponges). Unlike pre-fabricated embodiments or deployable effectors, *in situ* spun fibrous bodies emerge from environmental dynamics, thus the web is not an environment-referenced static object but functions as an interface that incorporates environmental information through interaction. This spiderweb-inspired, temporally and spatially scalable approach allows robots to extend, adjust, and repair their physical architecture in response to changing conditions, leading to robots that morph context-specific components and engage informational and material resources in the environment to tackle open-ended tasks beyond their initial physical architecture.

## 1 Introduction

While uncertainty challenges both robotic and biological systems, it enables emergent behaviors through robots’ immediate environmental interactions. Leveraging adaptive strategies of natural systems across micro- and macroenvironments promises robots with robust, task-agnostic adaptability. The prevalent robotics paradigm executes mobility tasks by planning and following movement trajectories (Mahmud et al., 2024; Nahavandi et al., 2025). Today’s robots can interact reasonably well with dynamic agents, such as other robots, humans, and vehicles, which is a significant achievement, even if such obstacles lie near the lower end of the dynamic complexity spectrum (Liu et al., 2024; Tang et al., 2025; Li et al., 2021). In more delicate and dynamic environments, predicting obstacle trajectories becomes computationally intractable (De Oliveira et al., 2025; Hausler et al., 2024). Even typical natural environments, such as wildlife habitats, are so dynamic that they are currently classified as extreme environments in machine learning and robotics (Polzin et al., 2025). Derived anthropogenic environments, such as disaster zones, pose similar challenges, with factors like unstable supports, occlusions, and deformable terrain further complicating robotic operations. Robots typically struggle to differentiate between genuine obstacles, such as branches or barbed wire, and harmless motions, such as swaying grass, due to the lack of a consistent frame of reference (Wang et al., 2024; Mohanan and Salgaonkar, 2022). Moreover, interpreting non-contact sensor data, such as visual and lidar inputs, to estimate obstacle path capabilities is slow and requires extensive contextual analysis. For instance, while a few straws may not halt a scout robot, the continuous and unpredictable movement of straws in the wind is computationally challenging. However, straws need to be detected to distinguish them from, e.g., glass shards that cannot be pushed aside in the same way as straws.

Bioinspiration suggests that operating in environments with the complexity and diversity of wild nature requires not only perceiving the environment as a reference frame but also leveraging it as an active resource. The progress in machine learning methods has made high-level strategies, such as task planning, relatively more advanced than deploying affordances in complex and dynamic environments. While robots with relatively simple bodies struggle with fine-grained control in dynamic environments, highly articulated, proprioceptive embodiments are better suited for low-level locomotion control but often struggle with strategic-level integration. Although high-level strategies continue to advance, there is a lack of low-level control systems capable of interfacing with them, limiting the integration of strategic planning with multiscale environmental affordances. A new, context-independent embodiment-centric solution is therefore needed, one that delegates computationally intensive, dynamic interaction and resource allocation tasks to the physical layer, while preserving centralized strategic control.

Affordances are universally used in mobile robotics to recognize and utilize the opportunities their environment presents for task performance. Robots can already identify affordances, typically by finding target objects in clutter and estimating properties like weight, hardness, or environmental traversability, often through the contextual analysis of visual data (Loeb, 2022; Apicella et al., 2025). Affordances grounded in bodily movement and physical contact are less common (Barsalou, 2007); however, the use of haptic data is gaining traction in soft robotics (Lovett et al., 2025). Distributed and grounded affordances are expressed primarily in the context of compliant interactions, in which a part of the robot, such as a gripper or other traction surface, deforms locally in response to the object’s morphology (Yasuda et al., 2021). Although soft gripping shows great commercial utility, the proportion of affordances currently used in robotics compared to those available to and used by organisms is very limited. Concurrently, physical groundedness remains an ongoing challenge in embodied AI, leading to affordance errors known as hallucinations (Yifan et al., 2025), which motivates the need to engage broader affordances to cope with real-world variability while still remaining within computational budget.

In tackling environmental uncertainty, bioinspired approaches have been fruitful. For example, plants and fungi (Russo et al., 2023) have inspired robots to move in and adapt to complex environments by growing (Mazzolai et al., 2020). Robots inspired by plant roots (Sadeghi et al., 2014) and climbing plants (Del Dottore et al., 2024) adapt to their environment by adding material to robot’s apical tip of the robot. Eversion robots, another strategy (Qin et al., 2025; Hawkes et al., 2017; Greer et al., 2020), add new material by unfolding from the base for the structure to grow. Recently, we presented a strategy for the robot to grow by spinning new components, thereby forming a gradual and continuously expanding interface between the robot and its environment (Vihmar et al., 2025). In these growing strategies, the physical interaction between the robot and its environment plays a major role in determining the final shape and position of the robot or its effector. Thus, the growth strategy has revealed the benefits of decentralized control and autonomous decision-making. The research on growing robots has, to date, focused on reactive action in morphologically complex, deformable, and granular environments. In contrast, the capability to operate within continuously moving environmental structures and the potential role of such motion in robots’ adaptation and performance have attracted little attention.

Spiders’ ability to spin various types of silk with different functionalities (Blackledge et al., 2012; Mariano-Martins et al., 2020) demonstrate another evolutionary strategy for adaptation and survival (Vollrath and Selden, 2007). The co-evolution of their bodies and silk production capabilities demonstrates elegant integration between body, material, and task, enabling interaction with their environment both spatially and temporally (Su and Buehler, 2016). Webs encode information about both the spider and the environment, inspiring robotics as context-responsive and affordance-driven models for embodiments. The versatile architectures of different spider webs have found interest as a source of inspiration for engineering (Mu et al., 2020; Su et al., 2021), structural design fabrication (Yang et al., 2022; Lu et al., 2023) and as a morphological computer (Hauser et al., 2014). Understanding spider webs can guide us toward design strategies that use path-finding and optimized construction to create complex, adaptive structures that are more responsive to their surroundings (Vollrath and Krink, 2020) even at an architectural scale (Kayser et al., 2018; Dall’Asta and Di Marco, 2025), which would not be feasible through prefabrication techniques.

**FIGURE 1:**
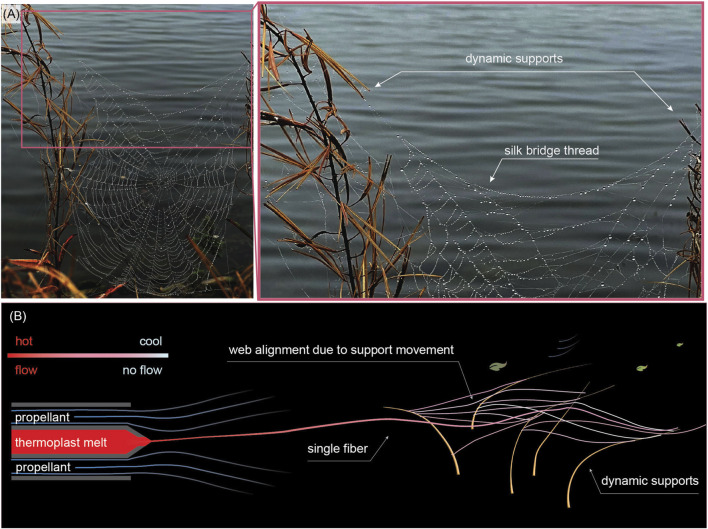
Spiderweb-inspired approach for tasks in a dynamic environment: from bioinspiration to implementation. **(A)** Photograph of a representative spider orb web (close-up on the right) anchored to wind-swaying plants during spinning and operation, illustrating architected interfaces to a dynamic environment. **(B)** Conceptual diagram of melt blow spinning to dynamic supports, with fibers shear-formed and propelled towards the supports by airflow. The red hue represents the temperature-dependent viscosity: the fiber exits the spinneret as a melt and solidifies into a web, depositing on the support while still capable of viscous rearrangement.

To our knowledge, the fabrication of web structures in dynamic environments remains unexplored in literature. From the embodiment adaptation perspective, the remarkable extensibility of spider silk enables the construction of webs into dynamic environments without compromising structural integrity or functional quality (Vepari and Kaplan, 2007; Mulder et al., 2021). The dynamics of the substrate may even offer an opportunistic advantage: building between fluctuating supports is demanding but highly rewarding for a spider (Brunetta and Craig, 2010) as having traps in spacious areas with ample airflow and access to insect pathways increases the likelihood of catching a generous amount of prey (Tadashi, 1997; Zschokke et al., 2006). Acceptance of dynamic supports may evidence a broader affordance benefit of contextual and transient embodiments. It departs from the uniformity and repeatability requirements inherent to engineering. The isotropic properties and unique pattern of the webs are both informed by the dynamic environment, in which they are situated, and may play a role in the contextual action. Figure 1A shows representative spiderwebs attached to highly dynamic structures. This observation can guide us closer to creating affordance-driven soft robots that can effectively deploy their desired functional embodiments into dynamic environments, utilizing spatial and temporal allowances and integration strategies (Fang et al., 2022). The effective use of the temporal environment promises a new modality of physical grounding for embodied AI.

We approached affordance broadly, considering a wide range of morphological and kinematic parameters, with the embodiment serving as the mechanism through which these affordances are realized. We are not treating the environment as an obstacle to be avoided or bypassed, but seeing it as a resource. Affordances are contextualized centrally and executed locally through sequential thermoplastic solidification primitives. Web spinning is inherently dynamic; while Su et al. (2021) have reported spiderweb construction phases, to our knowledge, there are no reports on how support movements during construction influence web morphology or how the gradual formation process can contribute to performance in a robotic context. To better understand affordance-driven embodiments, we blow-spun artificial spider webs from thermoplastic polymer on dynamically moving supports (Figure 1B). By deploying melt spinning, the thermoplastic fibers land on the supports while still allowing for viscous rearrangement, allowing them to arrange according to the movement of the support. This process is inherently emergent; the web morphology arises from the interaction between the spinning jet, environmental forces like airflow, and the location of nearby supports. While the state of the art relies on a single agent (such as the apex of a growing robot) determining the outcome, we utilize effectively thousands of fibers interacting locally, providing a statistical dimension on interactions.

In this work, we focus solely on the distributed part and test unchanged interaction physics across tasks with very different morphological characteristics, material properties, and dynamics, yet relevant to in-environment operation.

The contextual layer is enacted by a human operator controlling the spinneret. The operator controls only limited functions, such as the spinning angle and distance, to steer the process towards a self-set global objective (the level of automation success using physical AI), but individual fiber placement is not centrally controlled. Conversely, the fiber placement primitives are not informed of the global objective, e.g., they cannot distinguish whether it is engaged within a mobility or sampling task.

## 2 Methods

### 2.1 Melt blow spinning (MBS)

Ethylene vinyl acetate (EVA) based hot melt adhesive (Pattex) was used as received as the spinning medium. A custom spinneret featured a single concentric nozzle. EVA pieces were inserted into a centrally-located, temperature-controlled cylindrical chamber (∼10 cm^3^) and heated above their melting point (180 °C). Compressed air was applied to the chamber to drive the polymer melt through a nozzle on the opposite side. A foot pedal toggled the chamber between extrusion (isobaric) and no-extrusion (isovolumetric) modes. The spinneret nozzle was 3D-printed from 316L stainless steel (by JLC3DP). It featured a central polymer melt channel, lined with a stainless steel syringe needle (17G; internal diameter: 1.07 mm) to ensure surface smoothness, and surrounded by a concentric propellant chamber. Compressed air was used as a propellant and was heated by passing through a coiled heat exchanger, externally warmed with a variable-temperature hot air gun (up to ∼500 °C) to reach the desired propellant temperature at the nozzle. Independent pressure regulators were used to control the melt chamber and propellant pressures. Two thermocouples, one attached to the nozzle and the other to the propellant inlet, were used to monitor and adjust the process parameters.

Spinning was performed at nozzle temperatures ranging from 65 °C to 170 °C. Unless otherwise specified, a carrier gas pressure of 0.05 bar (measured at the spinneret inlet) and the extrusion pressure of 0.5 bar were used. The spinneret nozzle was positioned approximately 30 cm from the web at an angle of ∼40°.

### 2.2 Static web supports

Polymethyl methacrylate aperture plates with diameters of 20, 40, and 60 mm were fabricated using a laser cutter and secured in a bench vise during spinning. A wooden frame was fabricated out of a 16 × 9 mm profile and had an inner dimension of 1,005 mm (the extra 5 mm allowed for minimal frame deformation due to web contraction force).

### 2.3 Dynamical web supports

A cylindrical wooden rod (diameter: 4 mm) was used as support material. One rod was fixed stationary, and the second one was attached to a linear stage actuated using a stepper motor in a triangular pattern of 50-s period.

### 2.4 Web morphological characterization

Fiber diameter distributions were measured using the ImageJ software plugin DiameterJ, based on images obtained using a Di-Li 900-T optical microscope.

Fiber orientation distributions were measured using the ImageJ plugin OrientationJ. The webs were segmented into areas and photographed in 1:1 macro using a SONY FX30 camera. Visually distinct, high-fiber-density areas resulting from web partial ruptures were excluded. For web density distribution, 12 local distributions of non-overlapping 3.65-cm^2^ areas were averaged to calculate the standard deviation.

### 2.5 Tensile tests

AEL-A 100 Motorized Test Stand was used to measure mechanical characteristics. Rectangular cross-section rods made of plexiglass were prepared by laser cutting in pairs. The pulling speed was 2 cm s^−1^.

### 2.6 Demonstration experiments props

Sheep’s fescue (Festuca ovina) was glued to plexiglass strips at a uniform spacing. The strips were then mounted in parallel in extruded aluminum profile slots, allowing for linear movement. The strips were manually moved in a random pattern to mimic wind-driven swaying. Toy robots (wheeled and based on vibration acting on bristles) were used. Toy robots were released onto a 740 × 600 mm table enclosed by raised edges to prevent escape. Balance weights ranging from 1 to 100 g were used as models for granular materials. A 5 × 5 × 1-cm^3^ polyurethane sponge was immersed in vegetable oil (Oliva) as a model of an energy-rich resource.

## 3 Results

### 3.1 Morphology of substrate-free melt-blown spun web

Transition from spinning nonwoven mats on a substrate to direct spinning of substrate-free webs imposes new requirements on the spinning procedure. For a planar or cylindrical substrate, the spinneret-to-substrate distance and propellant flow profiles can be uniformly tuned. Additionally, the fiber-to-fiber intersections are often bonded, or the bond is strengthened post-spinning by adhesive immersion, e.g., using styrene-butadiene rubber. These steps ensure that the resulting non-woven strength is limited by the fibers themselves, not their interjunctions. Non-wovens could also be realized without bonding - in this case, the fibers form a web by physical fiber entanglement or high surface energy attraction (Russell, 2022.). In substrate-free direct web spinning, post-spinning adhesive application is hardly practical, especially in the dynamic case, where the eventual web forms as a result of web deformation during spinning. Nevertheless, post-spinning application of silicone elastomer was demonstrated in our previous work (Vihmar et al., 2025). Physical entanglement without adhesion shows more promise, as accelerated fibers can exhibit a richer variety of looping modes with previously deposited fibers, especially with the high-aspect-ratio supports in the environment. In this case, fiber-fiber slippage can be accepted, but fiber-to-fiber anchoring introduces a second level of stochasticity in web formation, which typically leads to areas that behave like a collection of loose fibers rather than a uniform web. Currently, there is no theory to predict or engage entanglement-based webs. The last, most straightforward, and thus most promising option is facilitating firm fiber-fiber attachment. In our previous work, we used an inhibited solvent evaporation in solution-blow-spinning that formed ‘islands’ spanning not just intersections in contact between two crossing fibers, but spanning several fibers, sometimes also including the support (Vihmar et al., 2025). As a result, a connected-island morphology was reached. In the dynamic case, we predicted that individual junction attachments are more practical than islands, as they allow more modes of fiber rearrangement during dynamic interaction. However, the spinneret-web distance and aerodynamic conditions vary widely in dynamic webs; thus, it is more difficult to set spinning parameters to reliably achieve cross-fiber junctions.

**FIGURE 2:**
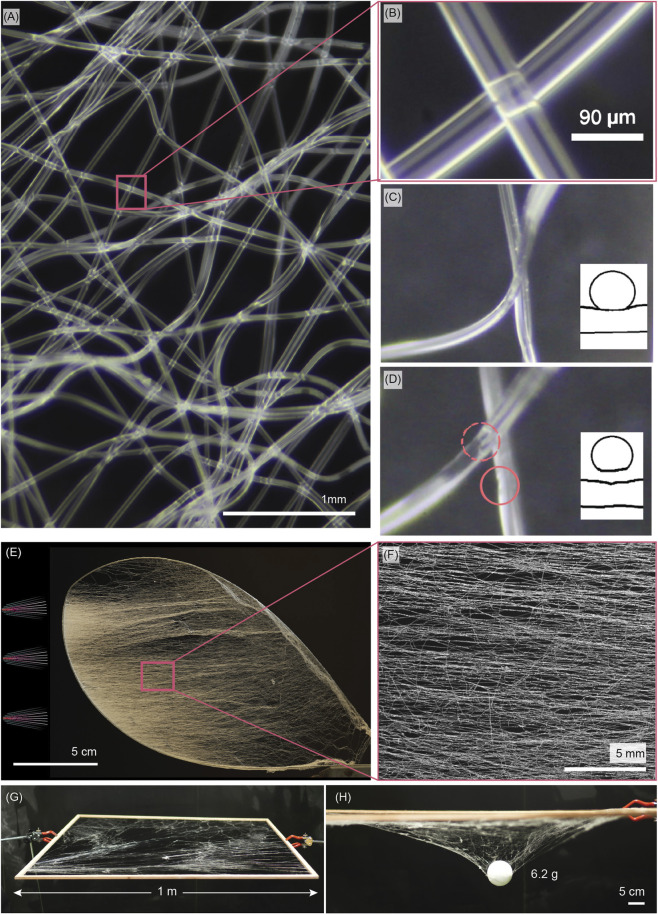
Substrate-free melt blow spun web morphology on static supports. **(A)** Macrophotograph of the web. **(B)** Zoom in to a representative fiber intersection, highlighting the top fiber acting as a cylindrical lens to observe the fiber below. **(C,D)** Pull test to test fiber intersection: a representative fiber intersection, **(C)** Before pull, and **(D)** After pull, showing small notches that evidence physical adhesion of warm top fiber during spinning. **(E)** A web spun at the lowest possible incidence angle onto a compliant Nylon loop support. **(F)** Macrophotograph showing fibers in the web predominantly aligned to the spin direction, but fibers at virtually all angles are seen. **(G,H)** Freestanding web spun on a 1-m square wooden frame. **(H)** Point-load test: a 6.2-g spherical expanded polystyrene weight suspended at midspan of the web, showing the sagging but intact network.

Figure 2A shows a typical close-up of an MBS web, spun substrate free between static supports. First, we observe that the fiber diameter is largely uniform across the observation area, indicating that most of the fiber geometry was defined shortly after exiting the spinneret, which contrasts with the broad diameter range of SBS, where the fibers are allowed to post-form and thin out over a longer period while already situated. The EVA fibers are optically transparent; thus, they act as infinitely long cylindrical lenses, allowing us to observe the cross-fiber junction by using the top fiber as a situated objective lens (in contact mode). In the typical junction, the bottom fiber is clearly observed (Figure 2B), implying that the fusion area is very localized. Nevertheless, the structure enacts a web without apparent detachment.

Based on the discussion above, we projected three hypotheses for web formation:

- The fibers are only entangled. The fibers solidify in the air and form webs through physical entanglement, but each intersection remains available for sliding. In a dynamic support case, this mode could allow for continuous web reconfiguration.
- As the EVA surface is macroscopically tacky, i.e., has a high surface energy, the web adhesion may be due to reversible physical adhesion between contacting fibers.
- The fibers reach the web partially solidified, and the crossing fibers are physically interlocked as a result of the high impact and shear forces during approach. The polymer in the fiber is still hot enough to allow for flow with respect to the incident fiber surface.

All three potential mechanisms are attractive in dynamic web scenarios, as they distribute local loads to a larger number of fibers.

To verify the dominant attachment mode, we pulled individual fibers using a needle tip under a microscope and tracked the attachment point. Indeed, the fiber crossing did not slide, disproving version A. As detached, the crossing remained detached, invalidating version B. Although the cylindrical lens setup suggested only a touch contact, the rupture point was visible as a small notch, shown in Figure 2D, thus validating case C being the dominant mechanism. As the junction was optically indistinguishable (Figure 2C) prior to rupture, this indicates that flow rearrangement and strong physical bonding were achieved, albeit very localized (approximately a few hundred μm^2^).

In our experiment on a single junction, the junction always ruptured before the fiber. This, however, does not imply the web strength is limited by junction strength, as junctions form between virtually all fiber crossings, and the cumulative strength may surpass that of one of the fibers. Figure 2E shows a substrate-free web spun at a very low incidence angle (approximately 10°), forming a visually easily distinguishable dominant fiber orientation aligned with the spinning direction. However, a close-up in Figure 2F confirms the presence of fibers at all orientations, including at a right angle with respect to the spinning direction. As each crossing creates an attachment point, a web that holds its shape also forms at an extremely low incidence angle.

Next, to understand the practical spatial limits of unsupported webs and any upper threshold in size, we spun a planar unsupported web between a 1-m^2^ frame, as shown in Figure 2G. Such a scale is relevant for, e.g., wildlife habitats that need to be monitored. In the experiment, the frame was horizontal. Covering a square meter scale frame required repositioning of the spinneret across the frame perimeter; however, the spinneret was, at all times, outside the frame vertical projection. Previously, we reported unsupported 0.038-m^2^ webs (Vihmar et al., 2025); however, we were under the impression that the size limitation applied only to our implementation, not to the process itself. Indeed, the 1-m^2^ web formed taut without showing scalability limits. As the spinning distance exceeded 0.5 m, the fiber flight time provided greater cooling, as evidenced by slightly reduced attachment to the web; however, the resulting web remained cohesive. Despite the web’s high stretchability, it still supported a load of 6.3 g (Figure 2H). The web conformed under the suspended load, further confirming the potential of using local affordances.

### 3.2 Spatial and morphological scalability of webs

**FIGURE 3:**
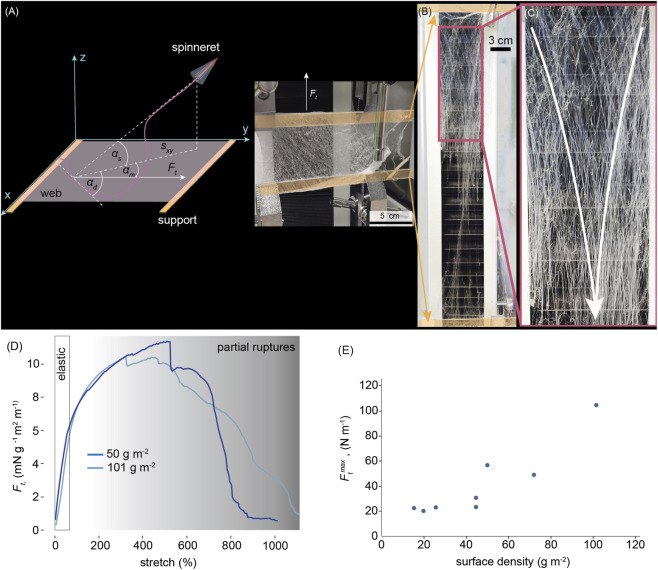
Mechanical properties of melt-blow-spun web. **(A)** Schematic (left) and photograph (right) of the tensile test set-up (left); **(B)** The web stretched to 1,000%, showing few intact fibers. **(C)** Close-up of a web stretched to its limit. White arrows indicate preferred fiber alignment directions when stretched during the tensile test. **(D)** Tensile tests of different-density webs. **(E)** Web strength-density dependence, n = 1.

To better understand web behavior at high deformations, we stretched uniaxially webs spun between two static supports, as shown in Figure 3A. As expected, the web with free length *l* failed gradually when stretched to the final distance *l’*, with some fibers still connecting the supports at stretch *λ* = *l’*/*l* = 1,000% (Figures 3B,C). This extreme *λ* supports the roles of a broad range of deposition angles and fiber alignment in stretch. The elastic range of *λ* is approximately 80%, as shown in Figure 3D, and is invariant with respect to web density. Thus, we expect the fibers to primarily orient and store elastic energy up to approximately *λ* = 80%, above which fiber crossings begin to rupture. Gradual rupture of fiber junctions dissipates energy and increases web toughness. Even though a single fiber cannot withstand a *λ* = 1,000%, the web retains a remnant connection even at such an extreme *λ*.

In depositing each new fiber to form a web (in a relatively low-density web), the number of fiber-to-fiber interconnections increases exponentially, as the new hot fiber makes interconnections to each fiber it crosses. However, web tensile strength was found to be nearly proportional to density (Figure 3E), confirming that fiber rearrangement, reorientation, and eventually gradual rupture, rather than intersection failure, were the mechanisms of web failure.

### 3.3 Dynamic supports

In the mechanical characterization above, the web was first spun between static supports and then stretched. But how does the web morphology differ in a web that is in continuous movement during spinning? To understand the effect of dynamic supports on *in situ* web morphological development, we spun a web between two horizontal supports (wooden rods). The spinneret was also fixed in a stationary position, eliminating any human operator bias.

**FIGURE 4:**
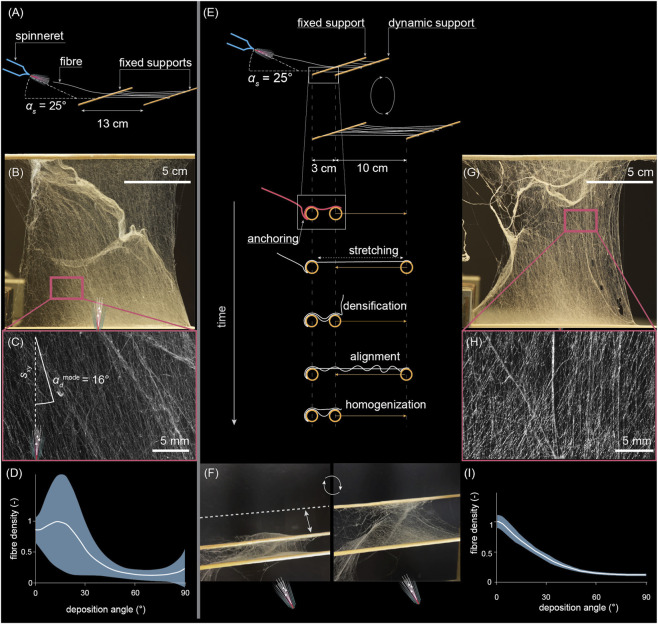
Contribution of support movement on web morphology. **(A–D)** Static support. **(A)** setup; **(B)** resulting web; **(C)** zoom-in of the web, demonstrating the α_d_^mode^. **(D)** Angular distribution of fiber orientation in the web. **(E–I)** Dynamic support **(E)** setup; **(F)** photograph of the web at extreme support positions during spinning. **(G)** Resulting web. **(H)** Zoom-in of the web. **(I)** Angular distribution of fiber orientation in the web. Blue corridors in **(D,I)** represent the standard deviation.

First, the two supports were both stationary at *l’* = *l* = 130 mm (Figure 4A). The resulting web, formed as a result of stochastic anchoring involving random web partial ruptures, is evidenced by pronounced denser areas, as shown in Figure 4B. The zoom-in micrograph of a representative area in Figure 4C shows deposition angle (*α*_*d*_) with a peak of 15.5° relative to the most prevalent fibre direction. However, the pronounced fiber direction did not exactly coincide due to local aerodynamic interactions and partial ruptures. By averaging fiber directionality across the web, as shown in Figure 4D, all deposition angles are expectedly present; however, the local fiber orientation varied drastically, as evidenced by a very wide standard deviation corridor, especially within the 0°–30° deposition angle range (with respect to supports), but also fiber distribution parallel to supports (90° deposition angle) varied substantially. As a key takeaway, even the morphologically simplest supports result in a spatially highly heterogeneous web, and heterogeneity and a stochastic formation process are crucial for utilizing affordances.

Next, we kept one support stationary while the other was attached to a horizontal linear stage actuated in a triangular pattern. The rod distance and web length, accordingly, changed continuously and drastically, ranging from *l* = 30 mm to *l’* = 130 mm. This movement corresponds to a *λ* = 433%, which, according to tensiometry data in Figure 3D, corresponds to the ultimate tensile strength (at approximately *λ* = 400–500%). This stretch is definitely above the elastic range, also confirmed by Figure 3D. However, a dynamic support engages diverse mechanisms that account for stretch in the web morphology; thus, data from an already spun nonwoven does not directly transfer to dynamic spinning. Figure 4E sketches the web formation during dynamic spinning. During each stretch, the fibers in the web were subjected to alignment, stretch (moderately above the elastic range), and even partial ruptures. In the release phase, the stretched web rearranged again. The newly deposited fibers during the stretch phase were then locally slacked by utilizing the stored elastic energy in the returning fibers. The slackened fibers were thereafter available for a longer stretch in the next cycle. The stretch-release cycles were repeated (38 cycles) during continuous spinning, indicating that the web can now engage in more diverse unpacking, alignment, and rupture mechanisms. Figure 4F shows a snapshot of the web during spinning in extreme positions. The overall web morphology, as shown in Figure 4G, was visually surprisingly similar to that between stationary supports (Figure 4B), with densified areas of comparable amount. However, the zoom-in (Figure 4H) reveals pronounced directionality that corresponds to support movement and web stretch direction. Thus, the support dynamics and spinning conditions were reflected in web morphology. As a key takeaway, even with morphologically simple yet dynamic supports, the resulting web is informed by the support’s movement history. As one possible outcome, the ‘memory’ makes the web more prepared to further movement in the previously experienced direction. This is evidently the key to autonomous exploitation of environmental affordance without sensor-actuator feedback loops.

The angular distribution of fibers in the web spun between dynamic supports (Figure 4I) was expectedly primarily aligned with the direction of support movement (most fibers at 0°), but the variability across the web was surprisingly narrow compared to Figure 4D. This further supports the ‘memory’ effect in the web, as the most prominent feature in the web exactly coincided with its experience during formation.

### 3.4 Demonstration experiments

We showcased the use of *in situ* spinning for a representative selection of environmental affordance challenges, as illustrated in Figure 6: mobility in dynamic environments, immobilization of dynamic objects, sampling granular materials, and resourcing of materials, suggesting that the environment affects the web formation.

**FIGURE 5:**
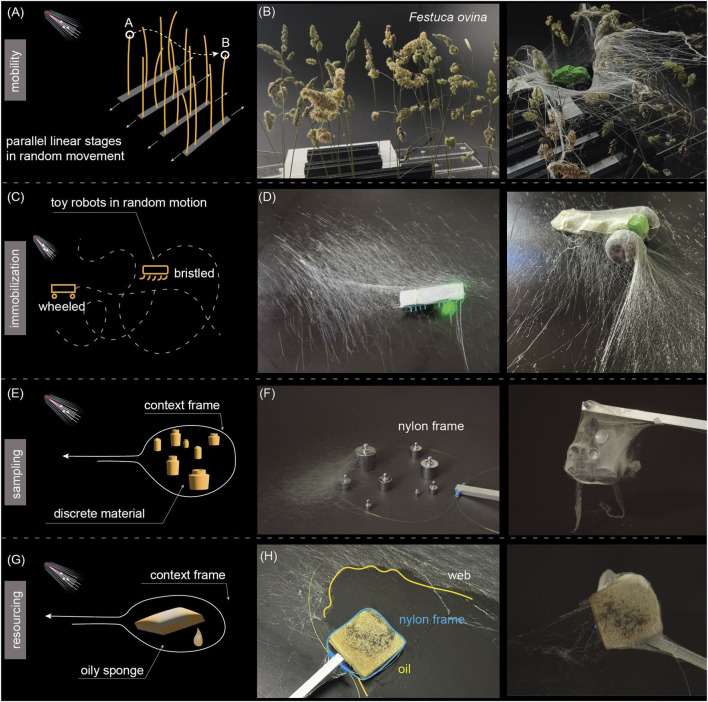
Demonstration experiments for the use of affordances by *in situ* spinning. **(A)** schematic of the mobility challenge setup. **(B)** Grass before (left) and after (right) spinning. A toy car placed on the web confirms that the web functions as a load distributor. **(C)** Immobilization challenge setup, featuring toy robots in rapid and random movement in an arena. **(D)** A bristled (left) and wheeled (right) toy robot immobilized by the spun fibers. **(E)** Sampling challenge setup, featuring balance weights as a model of granular material and a Nylon filament loop as a context frame. **(F)** Snapshot during spinning (left) and discrete material compacted by the web lifted from the arena with the help of the Nylon filament (right). **(G)** Resourcing challenge setup, featuring an oil-infused sponge. **(H)** Snapshot during spinning (left) and the sponge compartmentalized using the web and the Nylon filament (right).

First, we consider *mobility* challenges in a natural, delicate dynamic environment (Figure 5A). Grass (Festuca ovina) is enacted as a dynamic obstacle. To mimic stochastic movement, such as swaying in the wind, the grass stems were attached to parallel linear stages that were manually moved in a random pattern. The fibers were spun at approximately 40° from a fixed spinneret. The fibers adhered to the moving grass (Figure 5B, left and Supplementary Video S1). Each single fiber interacted with grass, whose movement stretched and aligned the fibers. This dynamic environment was co-designed through continuous interaction on the web. As the fibers were more oriented towards the movement direction, as shown in Figure 4I, the web gradually inhibited movement in the previous direction, effectively fixing the grass stems as a single agglomerate surface. Nevertheless, despite the morphological complexity and continuous movement of the supports, a relatively smooth web surface was formed. The web agglomerate effectively distributed the weight of a toy car placed on it, as shown in Figure 5B (right).

#### 3.4.1 Immobilization

Wheels are entanglement-prone. In our previous work (Vihmar et al., 2025), we demonstrated that spun webs form continuous, fused surfaces that accept wheeled platforms with wheel sizes as small as 6 mm. Moreover, because of their fused nature, the wheels could utilize friction and surface tackiness without entangling. The fused fiber architecture shown above (Figure 2A and Supplementary Video S2) confirms the case also with MBS webs, except for a quantitative difference in tackiness.

We hypothesize that entanglement-free behavior was due to locomotion on a fully developed web, and the behavior can be the opposite - inhibiting - when locomotion is attempted during spinning. To test this, fibers were spun toward an arena populated by battery-operated toy cars moving rapidly and randomly (Figure 5C). Indeed, the wheels of toy cars immediately entangled in the last deposited fiber and, via that, to the rest of the web, forming an agglomerate (Figure 5D, right). Vibration-based toy robots with angled-brush legs were also captured fast and efficiently (Figure 5D, left). Spider inspiration supports the immobilization function of webs.

#### 3.4.2 Sampling

Environmental affordances are also expressed in material substances that a robot could collect for analysis or use in its construction. The demonstrations above already confirm the aggregatory trend of web spinning; thus, we next showcased the capture of granular material (Figure 5E, and Supplementary Video S3). Our previous results (Vihmar et al., 2025) suggested modeling a gripper for pick-and-place tasks; however, we hypothesized that direct spinning on granular substances could form agglomerates that can be collected as a whole. As a model, we used varying sizes of steel balance weights: although each weight could be individually collected using a dedicated gripper, collecting all at once is also prohibitive for the human hand. Thus, *in situ* spinning is a promising alternative.

The first challenge in sampling is defining the objects of interest for collection within a particular context. For this, we first defined the ‘context frame’, or the perimeter of interaction, by placing a loop of Nylon filament around it (Figure 5E). Evidently, just tightening the filament ‘lasso’ would not allow for the collection of all items at once. However, by spinning on the granular substance surrounded by the ‘context frame’ (Figure 5F, left), the whole agglomerate was available for collection by lifting from the ends of the Nylon filament ‘context frame’ (Figure 5F, right). The filament also helped to support the weight of the agglomerate. A physical boundary around the targets defined the area of interaction necessary for executing the sampling task, as any fibers deposited elsewhere would rupture during the collection phase. This boundary focuses actions within a defined space. Importantly, such sampling is not a discrete event like conventional gripping, but a continuous process in which the sampled objects can vary in type or quantity.

Finally, we demonstrated a subset of sampling - *resourcing* an oil-infused sponge (Figure 5G). The rich chemical energy content of oil suggests scenarios for providing the robot with energy, paralleled by prey capture in spiders. Similar to granular samples, a Nylon loop ‘context frame’ was added to assist in compartmentalization (Figure 5H, left). Unlike the steel balance weights above, the sponge could be lassoed using the Nylon loop; however, the tightened loop would squeeze most of the oil out of the sponge during capture, compromising the objective. Here, the spun web helped compartmentalize the liquid-containing resource (Figure 5H, right), reducing liquid escape and facilitating possible integration with, for example, microbial fuel cells (Tsompanas et al., 2021) to access the chemical energy in the sample.

## 4 Discussion

### 4.1 Spinning as a task-independent operator

The fiber reacts autonomously and locally, without input from a central control system, to physical encounters, but it has no awareness of the global objective (the task). Thus, we foresee *in situ* spinning to increase the contribution of local processing in physical AI, but it is not to substitute the central awareness and strategic control layer. Autonomous fiber-level processing is to reduce central computation load, so that, e.g., movement in a complex environment is not via central path planning or obstacle classification. As a task-agnostic operator, web construction is determined solely by local affordances (e.g., support configuration and polymer rheology), emphasizing situatedness as a central feature of the control system: the outcome is specific to a particular time and space regardless of task context. Therefore, the central control layer introduces context (e.g., whether it is mobility or object handling?).

### 4.2 Taxonomy of spin-enabled interaction primitives

**FIGURE 6:**
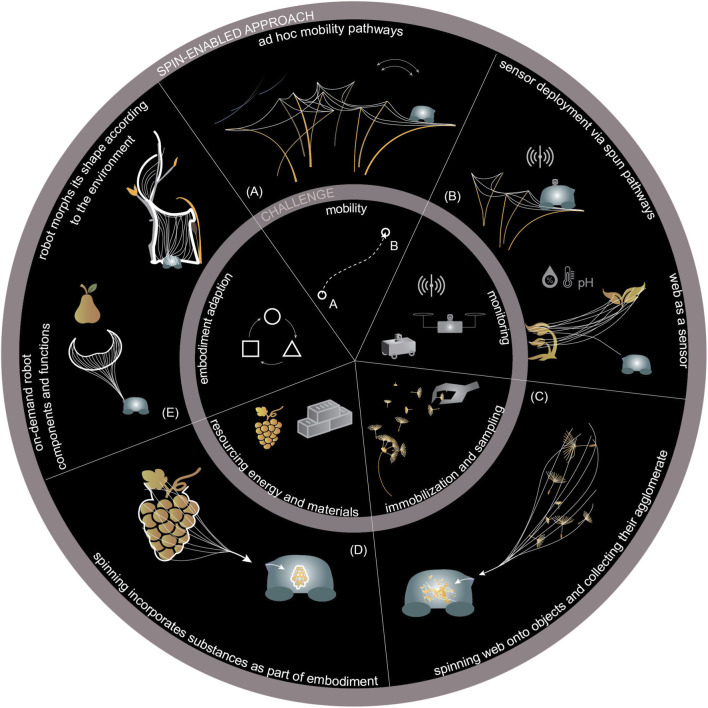
Concept map of affordance-driven on-demand spinning enabling robots to operate with on-demand embodiment in complex and dynamic environments, independent of preexisting infrastructure, creating temporary context-defined interfaces and components. The inner ring classifies core tasks, and the outer ring illustrates implementation pathways via contextual *in situ* spun embodiments.

To generalize the results, we first developed a taxonomy of partially overlapping interaction challenges and response scenarios in which *in situ* spinning could address the challenges, in which the web acts as a mediator between the robot and the environment (Figure 6):

- Mobility: forming pathways between moving supports for locomotion;
- Information acquisition: monitoring environmental conditions by deploying sensors via spun pathways or by using the web itself as a sensing structure;
- Sample acquisition: immobilizing and aggregating dynamic or granular samples through incremental spinning, enabling collection as static and cohesive agglomerates;
- Resource acquisition: incorporating objects from the environment into the robotic embodiment via incremental spinning, allowing their use as chemical energy sources or construction material;
- Environmental adaptation: using the environment to template the spun robot component, optimizing both morphological and contextual integration.

### 4.3 Dynamic environments favor an *in situ* construction strategy

To address embodiment-level autonomy in dynamic environments, our approach delegated interaction primitives to fiber-level interactions, equivalent to the tissue level in biological systems. Using simple input - pointing and toggling the spinneret - a variety of tasks across highly diverse contexts, including mobility and resource handling, were accomplished on supports ranging from swaying grass to granular or oil-perfused objects, supporting the idea of local action being task- and context-invariant. Dynamic supports enabled, rather than challenged, autonomous anisotropic structuring of the web, in contrast with closed-loop articulation-based systems, offering a more resource-conscious alternative. While universal grippers, often modeled after human hands, also aim to perform contextually diverse tasks, they currently struggle in dynamic scenarios due to the inherent complexity in continuously fusing proprioceptive feedback with task objectives to modulate gripper morphology. This work suggests that more involvement of local processes facilitates smoother global-local collaboration. While we operated the spinneret manually, we anticipate that contextual structuring (where a central controller gives the context and a local context-invariant layer executes it), rather than merely articulation, of embodiments will lead to more capable embodied AI.

In each of our demo examples, we deliberately did not implement on-board control of the spinneret. Instead, the spinneret was controlled with as few inputs as possible, at a predefined pressure and temperature, and the spinning direction was either static or manually adjusted. On one end, such spinneret direction control is straightforward to implement in control systems with visual feedback, as low-level interactions that would otherwise require substantial sensory data are fully delegated to the fiber level. Additionally, such central machine vision needs to allocate much fewer computational resources to non-task-essential aspects, such as swaying grass. On the other hand, the very same spinning setup enabled solving a wide range of contextually diverse tasks, ranging from sample collection to mobility. This manifests the use of environmental affordances and declares a sharp separation between the central contextual layer (what to do?) and its distributed implementation (how to do it? and who does it?).

### 4.4 Embodiment as an interface

The webs are not predesigned but context-shaped structures that encode environmental information directly into their morphology. The concept of robots extending their bodies *ad hoc* has been challenging due to a complex set of design parameters, one reason being the tendency to view the environment as an obstacle to navigate rather than a resource to utilize. Spiders, however, have evolved to act as environmental co-designers, continuously weaving webs that respond to situational demands through direct physical interaction. Similarly, in this work, we demonstrate this by spinning onto a dynamic support, which initiates constant fiber stretching, resulting in fiber alignment within the web, and the robot can shape its trajectory with preferred fiber alignment. The environment contributes to the web’s resulting morphology.

As the robot spins its web in the direction of its movement using the spinning device, it is expected that *α*_*d*_ mode aligns with *α*_*m*_ to achieve maximum strength in that direction. This demonstrates that the web spinning process is not entirely random but can be guided by anisotropic principles, without relying on any predefined models. *In situ* spinning enables the robot to adaptively reinforce its structure in real time, emphasizing organic growth and directionality without the constraints of a conventional model-driven path-planning approach.

This work demonstrates how an embodiment can gradually evolve into a negotiated interface, utilizing context-dependent spatial and temporal strategies. Obstacles are seen as environmental affordances and continuously considered and adjusted as part of shaping the embodiment. Webs can persist in dynamic conditions, as the fibers do not necessarily need to retain strength at elongation, but just retain general informedness of the previous conditions in the dynamic process via a few existing linkages.

We bring to attention that compartmentalization, i.e., the prevention of the uncontrolled spreading of system components into their environment, is also considered a criterion in popular definitions of life (Otto, 2021), and webs are particularly efficient at compartmentalization, as evidenced by the demonstration experiments above.

We expect future developments to improve understanding of the synergy between environmental/support dynamics and specific task contexts, such as how the informedness of prior deformations affects or improves tasks, similar to strain hardening. We also foresee the effect of local aerodynamic affordability on the specific mechanical behavior of the web, such as increased stretchability due to fiber packing.

Our long-term (10-year) moonshot is as follows: We conceptualize the physical body not as a static, bounded object, but as an emergent, gradual interface with the environment. Spatial and temporal fabrication enables the creation of soft robot embodiments that consider the environment as a resource rather than a reference point, demonstrating behavior similar to that of organisms. Web-based embodiments are not constrained by the task-fixed limitations typical of predefined fabrication and can adapt to *ad hoc* challenges. The new robots incorporate characteristics from the spiders’ use of webs, woven to “catch something in them of which they do not know exactly what it is, not exactly when it will come” (Rheinberger, 2012).

The spatiotemporally scalable spinning blurs the boundaries between the construction and operation of robots. Task-invariant spinning allows robots to consider virtually any object in the environment as a building block or a functionality, depending on centrally imposed goals. This process embraces latent intelligence, in which the robot’s embodiment executes environment-determined primitives that accept diverse tasks. By treating web-spinning as a model of emergent, situated, and distributed intelligence, it truly demonstrates context-aware adaptation.

## Data Availability

The original contributions presented in the study are included in the article/Supplementary Material, further inquiries can be directed to the corresponding author.
